# Power and Sample Size Determination in the Rasch Model: Evaluation of the Robustness of a Numerical Method to Non-Normality of the Latent Trait

**DOI:** 10.1371/journal.pone.0083652

**Published:** 2014-01-10

**Authors:** Alice Guilleux, Myriam Blanchin, Jean-Benoit Hardouin, Véronique Sébille

**Affiliations:** EA4275-SPHERE “Biostatistics, Pharmacoepidemiology and Subjective Measures in Health Sciences”, University of Nantes, France; VU University Amsterdam, Netherlands

## Abstract

Patient-reported outcomes (PRO) have gained importance in clinical and epidemiological research and aim at assessing quality of life, anxiety or fatigue for instance. Item Response Theory (IRT) models are increasingly used to validate and analyse PRO. Such models relate observed variables to a latent variable (unobservable variable) which is commonly assumed to be normally distributed. *A priori* sample size determination is important to obtain adequately powered studies to determine clinically important changes in PRO. In previous developments, the Raschpower method has been proposed for the determination of the power of the test of group effect for the comparison of PRO in cross-sectional studies with an IRT model, the Rasch model. The objective of this work was to evaluate the robustness of this method (which assumes a normal distribution for the latent variable) to violations of distributional assumption. The statistical power of the test of group effect was estimated by the empirical rejection rate in data sets simulated using a non-normally distributed latent variable. It was compared to the power obtained with the Raschpower method. In both cases, the data were analyzed using a latent regression Rasch model including a binary covariate for group effect. For all situations, both methods gave comparable results whatever the deviations from the model assumptions. Given the results, the Raschpower method seems to be robust to the non-normality of the latent trait for determining the power of the test of group effect.

## Introduction

The evaluation of perceived health outcomes or more generally patient-reported outcomes (PRO) is increasingly performed in different health areas. PRO combine self-reported information provided by the patient on his health or his treatment and aims at assessing his quality of life, anxiety or pain for instance. PRO differ from other health outcomes because these patient's characteristics cannot be directly measured such as overall survival for instance. These particular outcomes are usually evaluated using self-assessment questionnaires that are composed of a set of questions (called items) whose responses provided by the patients are analyzed.

Analysis of PRO can be based on two approaches: Classical Test Theory (CTT), or Item Response Theory (IRT) [1]. CTT relies on the observed scores (possibly weighted sum of patient item's responses) that are assumed to provide a good representation of a “true” score. IRT relies on an underlying response model relating the items responses to a latent unobservable variable, often called latent trait, usually assumed to follow a normal distribution and interpreted as a measure of the studied concept (quality of life, for example). IRT models are increasingly used to validate PRO instruments and to analyze these particular outcomes [2] [3] [4]. Moreover, amongst the large family of IRT models, the Rasch model [5] is often used for dichotomous items in health sciences. This model has interesting psychometric properties, in particular the specific objectivity property [6]. It involves that the patients can be objectively compared, that is to say independently of the questionnaire. Besides, the Rasch model presents several advantages such as the possibility to obtain a measure of the latent trait on an interval scale as well as the management of missing data and of possible floor and ceiling effects [7].

Despite the widespread use of PRO, the design and planning of studies, regarding careful *a priori* sample size and power determination, remain hardly ever provided. Furthermore, it has been stressed that many studies might not be adequately powered to determine clinically important changes in PRO [8] [9]. Specific sample size methodology is importantly needed for clinical research including PRO to avoid inadequately sized studies [10]. An inappropriate sample size determination could indeed lead to erroneous and uninformative conclusions or expose patients to inappropriate medical strategies.

The sample size for the comparison of a normally distributed endpoint in two independent groups can be computed using the usual formula conditionally on some assumed parameters values. Generally, the expected difference between mean values of the studied endpoint in the two groups (group effect γ) and the variance of the endpoint (σ^2^), often assumed to be equal in both groups, have to be defined. These parameters can be determined from a pilot study, the literature or experts opinions. Using these assumptions the sample size in each group can be determined for a given type I error and a given power.

The usual sample size formula could be used in the framework of IRT when the latent trait (endpoint) is assumed to be normally distributed. However, a previous study has shown that this widely-used formula was inadequate for IRT models because it leads to an underestimation of the required sample size [11]. This underestimation is closely related to the fact that the latent trait is an unobservable variable and that its estimation requires a model which creates uncertainty. Moreover, sample size determination is a key point for a rigorous planning in order to be able to determine an expected clinically relevant difference while controlling the type I and type II errors. Hence, an adaptation of the classical formula was required in order to offer a theoretical method for calculating the number of subjects for PRO studies [11] using IRT.

From this perspective, a method has been developed for power and sample size determination when an IRT model, the Rasch model, is intended to be used for analysis [12]. This method named Raschpower provides the power for a given sample size during the planning stage of a study in the framework of IRT.

This method has been validated under some conditions when all the assumptions of the underlying model were fulfilled. The aim is to study the impact of misspecifications of the distribution of the latent trait on the performance of the Raschpower method. The objective is to ensure that the Raschpower method produces reliable results when the required assumptions are not fulfilled. A simulation study is performed to assess whether the Raschpower method can still be used when the latent trait is not normally distributed.

## Method

### Rasch model

In IRT, the link between a latent trait (quality of life for example), and item parameters (items difficulties) is modelled. The probability that a person i (i = 1,…,N) responds x_ij_ to an item j (j = 1,…,J) is modelled with a logistic model depending on two parameters, the value of the latent trait of the person, θ_i_ and the difficulty of the item j, δ_j_. For a questionnaire composed of J dichotomous items answered by N patients, the Rasch model can be written as follows:
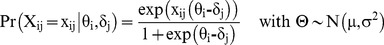
(1)where x_ij_ is a realization of the random variable X_ij_. θ_i_ are realizations of the random variable Θ, θ_1_, θ_2_,…, θ_N_ are mutually independent with a common underlying distribution which is generally assumed to be a normal distribution. In this case, the parameters of the Rasch model can be estimated by marginal maximum likelihood (MML) [13]. The Rasch model relies on three assumptions: i- unidimensionality: a unique latent variable explains the responses to the items; ii- monotonocity: the probability of a positive response to an item is a non-decreasing function of the latent variable; iii- local independence: given an individual, the item responses are independent of one another. A constraint has to be adopted to ensure the identifiability of the model: in the present paper, the mean of the latent trait (μ) is set to 0 [14] but the constraint can be put on either the mean of the latent trait or the mean of items difficulties.

### Determination of the power by the Raschpower method

To compare the means of the latent trait in two independent groups, we use the group effect (γ) which is the difference between the means of the latent trait in each group. The expected sample size is N_0_ in the first group and N_1_ in the second group. The latent regression Rasch model including a binary covariate for group effect is defined by the following:
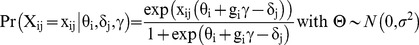
(2)To identify the model, the mean of the latent trait (μ) is 0 where μ is the mean between μ_0_ and μ_1_, each of them weighted by the sample sizes N_0_ and N_1_.

Consequently,
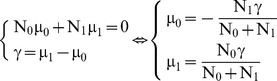
Θ is a random variable with normal distributions N
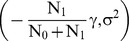
 and N
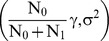
 in the first and the second group, respectively. Therefore g_i_ corresponds to 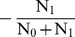
in the first group and to 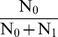
in the second group. The variance of the latent trait σ^2^ is assumed to be equal in the two groups.

The Wald test is used to compare the means of the latent trait in the two independent groups. The hypotheses for a two-sided test of comparison are defined as H_0_: γ = 0 against H_1_: γ≠0. To perform the test, an estimate Γ of γ and its variance are required and it is assumed that the test statistic 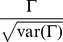
follows a normal distribution N(0,1) under H_0_. The patient's responses are also required to estimate the group effect's variance. At the planning stage, they are not known but a planning dataset can be determined conditionally on the assumed values for the sample size in each group (N_0_ and N_1_), the group effect (γ), the item difficulties (δ_j_) and the variance of the latent trait (σ^2^). For each possible response pattern (corresponding to a combination of responses of an individual to all items), the associated probability for each group is computed using the Rasch model. The expected frequency of each response pattern in each group is then determined. For dichotomous items, the number of response patterns corresponds to 2^J^ where J is the total number of items. The dataset composed of the expected frequencies associated to each response pattern is then analyzed with a latent regression Rasch model including a binary covariate for group effect. From this model, we estimate the group effect (γ) and its variance by the method of marginal maximum likelihood. To approximate the variance, we use the property of the Cramer-Rao (CR) bound which allows obtaining the lower bound of the variance of an unbiased estimator from the inverse of Fisher information. The expected power of the test of the group effect based on the Cramer-Rao bound can be approximated by [12]:
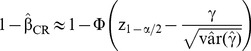
(3)where γ is assumed to take on positive values, z_1-α/2_ is the quantile of the standard normal distribution, Φ is the cumulative standard normal distribution function, and 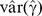 is evaluated using the Cramer-Rao bound.

### Simulation study

The Raschpower method assumes a normal distribution for the latent trait. To evaluate its robustness to departures from this normality assumption, the power determined with this method was compared to the power obtained using a simulation study, regarded as a reference. The data were simulated with a non-normal distribution for the latent trait and the empirical rejection rates obtained when fitting a model (assuming a normal distribution for the latent trait) to these simulated data was compared to the power obtained with the Raschpower method. The first step consisted in simulating data according to the parameters and underlying assumptions. The expected values for the parameters that are used at the planning stage of a study are the group effect (γ), the number of items (J), the item difficulties (δ_j_) but also the distribution of the latent trait. Two independent datasets (groups) were simulated. Each simulated scenario, corresponding to a combination of parameters, was replicated 1000 times. The simulated datasets were subsequently analyzed with a latent regression Rasch model including a binary covariate for group effect and assuming the normality assumption for the latent trait.

#### Simulated distributions of the latent trait

**Figure 1:**
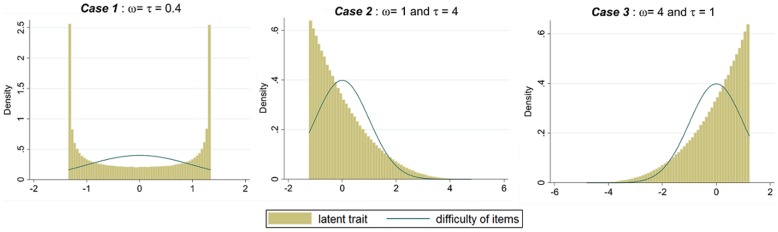
Distribution of the latent trait according to the different parameters of the beta distribution.

Data were simulated with a latent trait that followed a beta distribution depending on two parameters ω and τ. The beta distribution had different shapes depending on the value of these parameters which are both greater than zero (Figure 1). For example, when ω and τ were lower than 1, we obtained a U shaped distribution. If ω was set to 1 and τ was greater than 1 we obtained a L shaped distribution. Similarly, when ω was greater than 1 and τ was set to 1 we obtained a J shaped distribution.

These scenarios with different distributions of the latent trait reflect some specific situations:

The U shaped distribution means that the population is composed of individuals responding positively to most items and inversely, of individuals responding negatively to most items. The parameters of the beta distribution for this scenario have been fixed to ω = τ = 0.4.
The L shaped distribution corresponds to a situation in which individuals respond mostly negatively. The parameters of the beta distribution have been fixed to ω = 1 and τ = 4.
The J shaped distribution is the opposite situation where individuals respond mainly positively. In this case, the parameters of the beta distribution have been fixed to: ω = 4 and τ = 1.

#### Parameters of the simulation study

Several values of the simulated parameters were investigated. These values reflect cases often encountered in clinical and epidemiological research. The sample size in each group was: N_g_ = 50, 100, 200, 300, and 500. The number of items per questionnaire was: J = 5 and 10. The different values of group effects were: γ = 0, 0.2, 0.5, 0.8. The variance of the latent trait (σ^2^) was equal to 1. The mean of the latent trait (μ) was equal to 0. The item difficulty parameters corresponded to the percentiles of a normal distribution N(0,σ^2^). For J = 5, the item difficulties parameters were (δ_j_) = (−0.97, −0.43, 0, 0.44, 0.98) and for J = 10 (δ_j_) = (−1.33, −0.9, −0.6, −0.34, −0.11, 0.12, 0.36, 0.61, 0.92, 1.34). The different distributions of the latent trait were: L, J and U shaped. The combination of all parameters values led to 120 different cases and the simulations were replicated 1000 times.

#### Type I error and power determination in the simulation study

We estimated the group effect and its variance using the latent regression Rasch model including a binary covariate for the group effect on the simulated datasets. For the simulation study, the difficulty of items and the variance of the latent trait were fixed to their expected values. A Wald test was applied in order to estimate the type I error computed among the simulated datasets as the rejection rate of the null assumption (γ = 0) when the group effect was simulated at 0 (under H_0_). Similarly, the power was estimated among the simulated datasets as the rejection rate of the null assumption when the group effect was simulated at a value different from 0 (under H_1_).

### Evaluated criteria

To study the influence of the non-normality of the latent trait on the performance of the Raschpower method, several criteria were compared. The type I error (α) and their confidence interval were estimated using the simulated datasets. The power (1-β_S_) was obtained in the same way and compared to the power given by the Raschpower method based on the Cramer-Rao bound, (1-β_CR_). This latter is computed with the Raschpower module of Stata [12]. These comparisons allow determining whether the estimated power differ when the latent trait is not normally distributed. As the estimation of (1-β_CR_) is based on the estimated value of the variance of γ, a good estimation of the power requires a good estimation of this variance. Hence, the mean of the variance of the group effect in the simulations is compared with the estimated variance of the group effect using the Raschpower method. Along with these criteria, in the simulation study, the estimations of the group effect are studied to check that the estimated value is close to the simulated value for all cases. For the simulation study, the estimation of the group effect corresponds to the mean of the estimations obtained across the 1000 simulated datasets.

## Results

### Type I error of the test of group effect

Table 1 shows the empirical type I error for each of the 30 combinations of number of items (J), sample sizes (N_g_; g = 0,1), and distributions of the latent trait. All the type I errors are close to the expected value of 5%. Among the 30 estimates (when γ = 0), only two of the 95% confidence intervals do not contain the expected value of 5%. These cases are observed with N_g_ = 100, J = 10 and N_g_ = 200, J = 10 for a U shaped latent trait distribution (parameters of the beta distribution ω = τ = 0.4).

**Table 1:** Type I error and confidence intervals obtained using simulations according to the sample size (N_g_; g = 0,1), the number of items (J) and the distribution of the latent trait (Beta distribution).

J	Ng	U shaped	J shaped	L shaped
5	50	0.055 [0.042–0.071]	0.051 [0.038–0.067]	0.053 [0.040–0.069]
	100	0.057 [0.043–0.073]	0.049 [0.036–0.064]	0.048 [0.036–0.064]
	200	0.054 [0.041–0.070]	0.057 [0.043–0.073]	0.052 [0.039–0.068]
	300	0.053 [0.040–0.069]	0.054 [0.041–0.070]	0.055 [0.042–0.071]
	500	0.051 [0.038–0.067]	0.053 [0.040–0.069]	0.050 [0.037–0.065]
10	50	0.059 [0.045–0.075]	0.052 [0.039–0.068]	0.044 [0.032–0.059]
	100	0.066 [0.052–0.084]*	0.038 [0.027–0.058]	0.045 [0.033–0.060]
	200	0.066 [0.051–0.083]*	0.052 [0.039–0.068]	0.042 [0.030–0.056]
	300	0.058 [0.036–0.064]	0.053 [0.040–0.069]	0.049 [0.044–0.074]
	500	0.061 [0.047–0.078]	0.056 [0.043–0.072]	0.048 [0.036–0.063]

* Intervals not containing 5%.

### Estimation of the group effect

Table 2 presents the mean of the estimations of the group effect 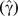 obtained using the simulated datasets according to the sample size (N_g_; g = 0,1), the number of items (J) and the different shapes of the distribution of the latent trait. For all distributions of the latent trait and parameter values, such as the sample size and the number of items, the group effect is correctly estimated. Estimations of the group effect 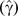 are close to their simulated values (γ). Among the 120 estimates, only three 95% confidence intervals do not contain the expected value of the group effect (γ), these results are not shown.

**Table 2:** Mean of the estimations of the group effect 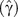 obtained using simulations according to the sample size (N_g_; g = 0,1), the number of items (J) and the distribution of the latent trait (Beta distribution).

		U shaped				J shaped				L shaped			
J	Ng	γ = 0	γ = 0.2	γ = 0.5	γ = 0.8	γ = 0	γ = 0.2	γ = 0.5	γ = 0.8	γ = 0	γ = 0.2	γ = 0.5	γ = 0.8
5	50	0.001	0.184	0.489	0.782	0.007	0.213	0.496	0.806	0.009	0.182	0.502	0.793
	100	0.004	0.198	0.495	0.796	0.010	0.206	0.499	0.809	0.011	0.202	0.492	0.798
	200	−0.002	0.199	0.486	0.786	3 10^−4^	0.198	0.498	0.807	0.002	0.195	0.508	0.802
	300	−0.003	0.200	0.489	0.786	−0.002	0.208	0.503	0.801	−0.003	0.198	0.509	0.800
	500	−0.004	0.196	0.497	0.789	−0.002	0.205	0.497	0.802	−0.004	0.198	0.499	0.810
10	50	−0.011	0.204	0.490	0.800	−0.001	0.193	0.502	0.806	−0.004	0.186	0.506	0.801
	100	0.005	0.203	0.501	0.797	−0.003	0.200	0.494	0.804	8 10^−5^	0.200	0.500	0.802
	200	−0.005	0.201	0.500	0.795	−0.001	0.193	0.501	0.810	−0.007	0.197	0.502	0.809
	300	0.001	0.196	0.497	0.796	−0.003	0.204	0.499	0.802	−0.002	0.203	0.497	0.797
	500	0.002	0.197	0.497	0.794	−0.002	0.204	0.503	0.799	4 10^−4^	0.198	0.500	0.799

### Estimation of the variance of the group effect

Table 3 presents the results of the estimation of the mean variance of the group effect (var_S_) using the simulated datasets, and the variance of the group effect obtained with Raschpower (var_CR_) for different values of the group effect (γ), the sample size in each group (N_g_; g = 0,1) and the number of items (J). These results are related to the case where the latent trait has a beta U shaped distribution.

**Table 3:** Estimation of the variance of the group effect using simulations (var_S_) and using the Raschpower method (var_CR_) according to the different values of group effect (γ), sample size in each group (N_g_; g = 0,1) and number of items (J).

		γ							
		0		0.2		0.5		0.8	
	Ng	var_S_	var_CR_	var_S_	var_CR_	var_S_	var_CR_	var_S_	var_CR_
J = 5	50	0.0821	0.0818	0.0822	0.0819	0.0825	0.0823	0.0831	0.0827
	100	0.0410	0.0409	0.0406	0.0409	0.0412	0.0411	0.0415	0.0414
	200	0.0205	0.0205	0.0205	0.0205	0.0206	0.0206	0.0207	0.0207
	300	0.0137	0.0136	0.0137	0.0136	0.0137	0.0137	0.0138	0.0138
	500	0.0082	0.0082	0.0082	0.0082	0.0082	0.0082	0.0083	0.0083
J = 10	50	0.0617	0.0604	0.0617	0.0606	0.0619	0.0613	0.0622	0.0639
	100	0.0308	0.0303	0.0309	0.0304	0.0309	0.0310	0.0311	0.0315
	200	0.0154	0.0152	0.0154	0.0153	0.0155	0.0155	0.0155	0.0156
	300	0.0103	0.0103	0.0103	0.0102	0.0103	0.0103	0.0104	0.0104
	500	0.0062	0.0062	0.0062	0.0062	0.0062	0.0062	0.0062	0.0062

U shaped distribution case for the latent trait.

The results show that, whatever the values of the parameters, the estimations of the variances of the group effect are close (between the simulations and the Raschpower method). The difference between the estimations of the variances is on average 8.75 10^−4^ and it fluctuates between −0.0017 (J = 10, N_g_ = 50, γ = 0.8) and 0.0013 (J = 10, N_g_ = 50, γ = 0). Among the expected effects, we find that the variance of the group effect decreases when the sample size (N_g_; g = 0,1) increases whereas it rises, but only slightly, when the group effect (γ) increases. Moreover, the variance of the group effect drops when the number of items (J) expands. Other simulation results obtained with L and J shaped distributions of the latent trait are similar, the variance estimates for the group effect obtained in the simulations are very close to those given by the Raschpower method (results shown Table S1).

### Power of the test of group effect

**Figure 2:**
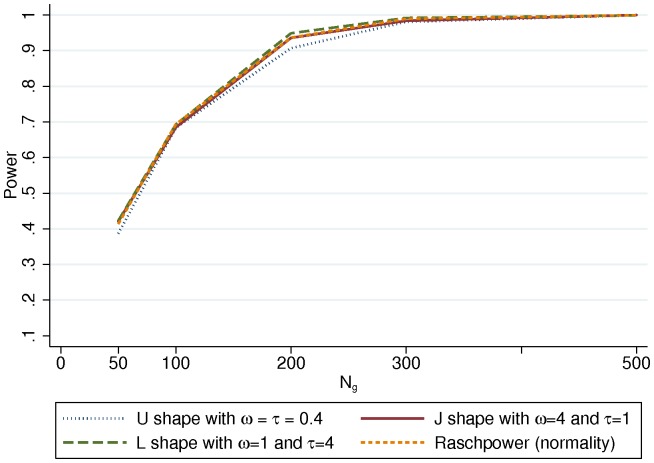
Power obtained using simulation and using the Raschpower method as a function of the sample size (N_g_; g = 0,1) with a group effect (γ) set at 0.5 and for 5 items (J).

Figure 2 shows the power obtained using the simulated datasets (for the U, J and L shaped distributions) and using Raschpower according to different sample sizes (N_g_; g = 0,1) in the case where the group effect (γ) is 0.5 and the number of items (J) is equal to 5. For all distributions of the latent trait (Normal or Beta), the power are close to each other. The difference between the powers obtained with the simulations and with Raschpower is about −0.006 on average and fluctuates between −0.03 (J = 5, γ = 0.5, N_g_ = 200 and U shaped distribution of the latent trait) and 0.012 (J = 5, γ = 0.5, Ng = 200 and L shaped distribution of the latent trait). As expected, for all 120 cases (results shown in Table S2), the power increases with the sample size (N_g_; g = 0,1), the group effect (γ) and the number of items (J). For example, with the Raschpower method using N_g_ = 100, with γ = 0.5 and J = 5, the power is 69.4% and it is 81.1% when J = 10.

## Discussion

The Raschpower method provides Rasch-based power determination for two-group cross-sectional comparisons when a Rasch model is intended to be used for analysing PRO data. It relies on some assumptions and in particular the normality of the latent trait distribution which might not be encountered in practice. The impact of a deviation from the normality assumption on the determination of the power of the test of group effect using the Raschpower method was studied using simulations. The power is a key point for the determination of sample size at planning stage for cross-sectional studies comparing two groups and it depends on two parameters: the group effect and its variance.

The results have shown that the powers estimated using either simulations (with a non-normal distribution of the latent trait) or the Raschpower method were very close. The violation of the assumption regarding the distribution of the latent trait had very little impact on the estimation of the variance of the group effect and thus on the power of the test of group effect. As expected, the power varied with different parameters; in particular, it increased when the number of items, the sample size and the group effect rose.

Some methodological choices can be discussed regarding both the distributions of the latent trait and items parameters. The robustness of the Raschpower method might be related to the fact that the distributions of the latent trait and of the items parameters were overlaid. Indeed, in this study, the items parameters were simulated as regularly distributed and adapted to a population with a latent trait distributed in the same range of values. This reflects a questionnaire that is neither too easy nor too difficult for patients with some balance of positive and negative responses. Moreover, the regularity of the distribution involves that the latent trait of individuals is evaluated with a similar precision all over its continuum. Finally, the choice of overlapping distributions avoids the presence of ceiling and floor effects. It has been shown in previous results [15] that the Raschpower method is valid when the items distribution is overlaid with the latent trait distribution.

Some other aspects related to the robustness of the Raschpower method could also be investigated. It could be interesting to study some parameters misspecifications, especially those that might affect the power of the test of group effect, such as the variance of the latent trait (σ^2^) for instance. The purpose would be to get more insight regarding the impact on the performance of the Raschpower method when the expected value of the parameter (fixed at planning stage) is different from the observed value on the data (analysis stage).

The violation of the assumption of normality of the latent trait does not impact the estimation of the variance of the group effect. The power is correctly estimated and the Raschpower method is robust for power analyses of PRO data analysed with a Rasch model. This issue of robustness to misspecification of the distribution of random effects has been approached in a more general way in generalized linear mixed models (GLMM) from which IRT models such as the Rasch model come from [16] [17] [18]. The consequences of misspecifying the random-effects distribution on the estimation and hypothesis testing in GLMM was studied, through simulations. Different distributions and variances of the random-effect were investigated. The results have shown that in the context of small variance and only one random-effect, the estimations were correct as well as the control of the type I and II errors for all simulated distributions. Moreover, the estimates of the fixed effects are much less sensitive to misspecification of the random effects distribution [19].

In the framework of our study, the Raschpower method and the Rasch model used in the simulation study were performed in the same conditions with small variance and only one random-effect. Furthermore, in simulations, the fixed effect (γ) was estimated without bias meaning that the estimation of the power of the group effect was likely not badly affected by this estimation. Thus, even though the analyses of the simulated datasets were based on a Rasch model assuming a normal distribution for the latent trait, the power estimated in those simulations, seems reliable and can be used as the reference for the comparison with the Raschpower method. Our results are consistent with results from Litière *et al.* and Fitzmaurice *et al* [16] [18]. These results bring new elements about the robustness of the Raschpower method and complete those of the literature on the robustness in GLMM. The Raschpower method seems to be robust to non-normality of the latent trait and the power and type I error are not affected by a misspecification of the distribution of the latent trait.

## Supporting Information

Table S1 **Estimation of the variance of the group effect using simulations (var_S_) and using the Raschpower method (var_CR_) according to different values of group effect (γ), sample size in each group (N_g_; g = 0,1) and number of items (J).** L and J shaped distributions case for the latent trait.
(DOC)

Table S2
**Power estimated by the Raschpower method (1-** 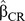 **) and using simulations (1-** 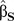 **) for the Wald test comparing the means of the latent trait in the two groups according to the values of the group effect (γ) the sample size (N_g_; g = 0,1) and the number of items (J).**
(DOC)
