# Supplementary material for: Power and Sample Size Determination in the Rasch Model: Evaluation of the Robustness of a Numerical Method to Non-Normality of the Latent Trait
Source: PLoS One. 2014 Jan 10;9(1):e83652. doi: 10.1371/journal.pone.0083652 (PMC3888396; doi:10.1371/journal.pone.0083652)
Supplement: Table S1 — Estimation of the variance of the group effect using simulations (varS) and using the Raschpower method (varCR) according to different values of group effect (γ), sample size in each group (Ng; g = 0,1) and number of items (J). L and J shaped distributions case for the latent trait. (DOC) [file pone.0083652.s001.doc]

|  |  | **L shaped distribution** | | | | | | | |
| --- | --- | --- | --- | --- | --- | --- | --- | --- | --- |
|  |  | γ | | | | | | | |
|  |  | 0 | | 0.2 | | 0.5 | | 0.8 | |
|  | Ng | var_S_ | var_CR_ | var_S_ | var_CR_ | var_S_ | var_CR_ | var_S_ | var_CR_ |
| J=5 | 50 | 0.0819 | 0.0818 | 0.0819 | 0.0819 | 0.0823 | 0.0823 | 0.0829 | 0.0827 |
|  | 100 | 0.0409 | 0.0409 | 0.0409 | 0.0409 | 0.0411 | 0.0411 | 0.0414 | 0.0414 |
|  | 200 | 0.0205 | 0.0205 | 0.0205 | 0.0205 | 0.0205 | 0.0206 | 0.0207 | 0.0207 |
|  | 300 | 0.0136 | 0.0136 | 0.0136 | 0.0136 | 0.0137 | 0.0137 | 0.0138 | 0.0138 |
|  | 500 | 0.0082 | 0.0082 | 0.0082 | 0.0082 | 0.0082 | 0.0082 | 0.0083 | 0.0083 |
| J=10 | 50 | 0.0615 | 0.0604 | 0.0615 | 0.0606 | 0.0617 | 0.0613 | 0.0620 | 0.0639 |
|  | 100 | 0.0307 | 0.0303 | 0.0307 | 0.0304 | 0.0308 | 0.0310 | 0.0310 | 0.0315 |
|  | 200 | 0.0154 | 0.0152 | 0.0154 | 0.0153 | 0.0154 | 0.0155 | 0.0155 | 0.0156 |
|  | 300 | 0.0102 | 0.0103 | 0.0102 | 0.0102 | 0.0103 | 0.0103 | 0.0103 | 0.0104 |
|  | 500 | 0.0061 | 0.0062 | 0.0061 | 0.0062 | 0.0062 | 0.0062 | 0.0062 | 0.0062 |
|  |  | **J shaped distribution** | | | | | | | |
|  |  | γ | | | | | | | |
|  |  | 0 | | 0.2 | | 0.5 | | 0.8 | |
|  | Ng | var_S_ | var_CR_ | var_S_ | var_CR_ | var_S_ | var_CR_ | var_S_ | var_CR_ |
| J=5 | 50 | 0.0819 | 0.0818 | 0.0819 | 0.0819 | 0.0823 | 0.0823 | 0.0829 | 0.0827 |
|  | 100 | 0.0409 | 0.0409 | 0.0409 | 0.0409 | 0.0411 | 0.0411 | 0.0414 | 0.0414 |
|  | 200 | 0.0204 | 0.0205 | 0.0205 | 0.0205 | 0.0205 | 0.0206 | 0.0207 | 0.0207 |
|  | 300 | 0.0136 | 0.0136 | 0.0136 | 0.0136 | 0.0137 | 0.0137 | 0.0138 | 0.0138 |
|  | 500 | 0.0082 | 0.0082 | 0.0082 | 0.0082 | 0.0082 | 0.0082 | 0.0083 | 0.0083 |
| J=10 | 50 | 0.0615 | 0.0604 | 0.0615 | 0.0606 | 0.0617 | 0.0613 | 0.0620 | 0.0639 |
|  | 100 | 0.0307 | 0.0303 | 0.0307 | 0.0304 | 0.0308 | 0.0310 | 0.0310 | 0.0315 |
|  | 200 | 0.0154 | 0.0152 | 0.0154 | 0.0153 | 0.0154 | 0.0155 | 0.0155 | 0.0156 |
|  | 300 | 0.0102 | 0.0103 | 0.0102 | 0.0102 | 0.0103 | 0.0103 | 0.0103 | 0.0104 |
|  | 500 | 0.0061 | 0.0062 | 0.0061 | 0.0062 | 0.0062 | 0.0062 | 0.0062 | 0.0062 |
