# Supplementary material for: Power and Sample Size Determination in the Rasch Model: Evaluation of the Robustness of a Numerical Method to Non-Normality of the Latent Trait
Source: PLoS One. 2014 Jan 10;9(1):e83652. doi: 10.1371/journal.pone.0083652 (PMC3888396; doi:10.1371/journal.pone.0083652)
Supplement: Table S2 — Power estimated by the Raschpower method (1- ) and using simulations (1- ) for the Wald test comparing the means of the latent trait in the two groups according to the values of the group effect (γ) the sample size (Ng; g = 0,1) and the number of items (J). (DOC) [file pone.0083652.s002.doc]

|  |  |  | 1-$\hat{\text{β}}_{\text{S}}$ | | |  | 1-$\hat{\text{β}}_{\text{CR}}$ |
| --- | --- | --- | --- | --- | --- | --- | --- |
| J | γ | N_g_ | U shaped | J shaped | L shaped |  |  |
| 5 | 0.2 | 50 | 0.097 | 0.112 | 0.105 |  | 0.104 |
|  |  | 100 | 0.169 | 0.178 | 0.158 |  | 0.166 |
|  |  | 200 | 0.286 | 0.271 | 0.264 |  | 0.287 |
|  |  | 300 | 0.402 | 0.429 | 0.393 |  | 0.402 |
|  |  | 500 | 0.566 | 0.617 | 0.592 |  | 0.599 |
|  | 0.5 | 50 | 0.387 | 0.421 | 0.423 |  | 0.414 |
|  |  | 100 | 0.684 | 0.685 | 0.693 |  | 0.694 |
|  |  | 200 | 0.907 | 0.936 | 0.949 |  | 0.937 |
|  |  | 300 | 0.981 | 0.984 | 0.992 |  | 1 |
|  |  | 500 | 1 | 1 | 0.999 |  | 1 |
|  | 0.8 | 50 | 0.760 | 0.795 | 0.790 |  | 0.794 |
|  |  | 100 | 0.976 | 0.979 | 0.980 |  | 0.976 |
|  |  | 200 | 1 | 1 | 1 |  | 1 |
|  |  | 300 | 1 | 1 | 1 |  | 1 |
|  |  | 500 | 1 | 1 | 1 |  | 1 |
| 10 | 0.2 | 50 | 0.135 | 0.119 | 0.118 |  | 0.126 |
|  |  | 100 | 0.208 | 0.214 | 0.207 |  | 0.208 |
|  |  | 200 | 0.383 | 0.342 | 0.354 |  | 0.365 |
|  |  | 300 | 0.492 | 0.526 | 0.522 |  | 0.506 |
|  |  | 500 | 0.719 | 0.759 | 0.711 |  | 0.722 |
|  | 0.5 | 50 | 0.514 | 0.512 | 0.526 |  | 0.524 |
|  |  | 100 | 0.802 | 0.807 | 0.819 |  | 0.811 |
|  |  | 200 | 0.973 | 0.983 | 0.982 |  | 0.980 |
|  |  | 300 | 1 | 0.999 | 0.998 |  | 0.999 |
|  |  | 500 | 1 | 1 | 1 |  | 1 |
|  | 0.8 | 50 | 0.887 | 0.919 | 0.897 |  | 0.886 |
|  |  | 100 | 0.991 | 0.997 | 0.992 |  | 0.995 |
|  |  | 200 | 1 | 1 | 1 |  | 1 |
|  |  | 300 | 1 | 1 | 1 |  | 1 |
|  |  | 500 | 1 | 1 | 1 |  | 1 |
